# Association between Endothelial Activation and Stress Index and mortality in diabetic nephropathy ICU patients: A retrospective cohort study

**DOI:** 10.1371/journal.pone.0329233

**Published:** 2025-08-14

**Authors:** Sheng Chen, Lin Guo, Xiaohan Ma, Shuaikang Wang, Junchao Wu, Lingling Wu, Ting Zhang, Hongjun Gao

**Affiliations:** 1 Graduate School, Guangxi University of Chinese Medicine, Nanning, Guangxi, China; 2 Ruikang Hospital, Guangxi University of Chinese Medicine, Nanning, Guangxi, China; Tehran University of Medical Sciences, IRAN, ISLAMIC REPUBLIC OF

## Abstract

**Background:**

Diabetic nephropathy (DN) is a serious complication of diabetes mellitus, often leading to poor outcomes in critically ill patients. Endothelial Activation and Stress Index (EASIX), a marker of endothelial dysfunction and systemic stress, has been associated with adverse outcomes in various diseases, but its role in predicting mortality in DN patients remains unclear.

**Methods:**

A retrospective cohort study was conducted using the MIMIC-IV database. A total of 1,260 critically ill DN patients were included and stratified into tertiles based on their EASIX scores. Kaplan-Meier survival analysis, Cox proportional hazard models, and restricted cubic spline regression were applied to evaluate the association between EASIX and 30- and 60-day all-cause mortality. Subgroup analyses were also performed to assess interactions with key patient characteristics.

**Results:**

Patients with higher EASIX scores had significantly increased ICU and in-hospital mortality rates. Cox regression analyses revealed that EASIX was an independent predictor of mortality after adjusting for age, sex, and comorbidities (HR: 1.14; 95% CI: 1.03–1.26; p = 0.01). Kaplan-Meier analysis showed significantly worse survival rates in the highest EASIX tertile. Subgroup analysis showed that higher EASIX scores were still associated with short-term survival in patients with DN in the presence of older age, male gender, and severe complications.

**Conclusion:**

Higher EASIX scores are associated with increased short-term mortality in critically ill DN patients, highlighting its value as a prognostic biomarker for risk stratification and personalized management. Further studies are needed to validate these findings and explore therapeutic interventions targeting endothelial dysfunction.

## 1. Introduction

Diabetic nephropathy (DN) is a major complication of diabetes mellitus, frequently resulting in end-stage renal disease and requiring renal replacement therapies such as dialysis or transplantation. The pathophysiology of DN involves intricate interactions between metabolic and hemodynamic factors, primarily driven by chronic hyperglycemia. Clinically, DN is marked by albuminuria, a decline in glomerular filtration rate, and progressive kidney damage. However, recent studies have challenged the traditional view of DN, revealing that non-proteinuric forms of DN are more common than previously believed. This underscores the need for updated diagnostic criteria and biomarkers to detect early renal dysfunction in diabetic patients [1–3].

The Endothelial Activation and Stress Index (EASIX) is a clinical tool that assesses endothelial dysfunction and stress, both of which are pivotal in the development of cardiovascular diseases. Endothelial dysfunction involves decreased nitric oxide production, increased oxidative stress, and activated inflammatory pathways, contributing to conditions like atherosclerosis and hypertension [4,5]. It is a well-established predictor of cardiovascular events, with markers such as soluble intercellular adhesion molecule-1 and soluble vascular cell adhesion molecule-1 elevated in affected individuals [6]. Additionally, oxidative stress markers, such as malondialdehyde and oxidized low-density lipoprotein, are often elevated, further heightening cardiovascular risk [7]. The EASIX score, which combines endothelial activation and stress markers, has been used to predict clinical outcomes. For example, in patients receiving chimeric antigen receptor T cell therapy, a higher EASIX score correlates with an increased risk of cytokine release syndrome and consumptive coagulopathy, both associated with endothelial dysfunction [8]. This underscores the importance of monitoring endothelial health, particularly during intensive treatments. Moreover, oxidative stress can activate the endoplasmic reticulum stress pathway, exacerbating endothelial dysfunction by promoting inflammation and apoptosis [9].

Endothelial activation is central to the development of various cardiovascular diseases and is strongly linked to inflammation. The EASIX is a biomarker studied in traumatic brain injury, where it predicts mortality outcomes. Calculated from lactate dehydrogenase, creatinine, and platelet levels, elevated EASIX reflects endothelial stress and activation, often accompanied by inflammation [10]. Recent studies have also examined inflammation’s role in DN progression. Inflammatory cells, including macrophages and T-lymphocytes, contribute to DN, suggesting that targeting inflammatory pathways could lead to new treatments. Identifying specific inflammatory cytokines and stress-activated protein kinases in DN offers potential targets for pharmacological intervention [10,11].

To date, no studies have explored the relationship between the EASIX and DN. Thus, this study aimed to examine the association between EASIX and 30- and 60-day all-cause mortality in patients with DN. We hypothesized that higher EASIX scores would correlate with an increased risk of mortality in these patients.

## 2. Materials

### 2.1. Study population

This retrospective study utilized health data from the Medical Information Mart for Intensive Care (MIMIC)-IV (version 3.1) database, a comprehensive, high-quality medical record repository managed by the Computational Physiology Laboratory at the Massachusetts Institute of Technology. The database contains detailed records of patients admitted to the intensive care unit at Beth Israel Deaconess Medical Center [12].

The studies involving human participants were reviewed and approved by MIMIC-IV databases were approved by the institutional review boards of the Massachusetts Institute of Technology and Beth Israel Deaconess Medical Center. Written informed consent for participation was not required for this study in accordance with the national legislation and the institutional requirements.

In accordance with the established protocols and ethical guidelines for the use of the MIMIC databases, one of the authors of this study, Sheng Chen, successfully completed a comprehensive human subject research training course, which was designed to ensure adherence to ethical standards when handling patient data. This course, identified by the record ID 66963781, provided the necessary educational foundation on human subject research ethics and best practices in data handling.

**Fig 1:**
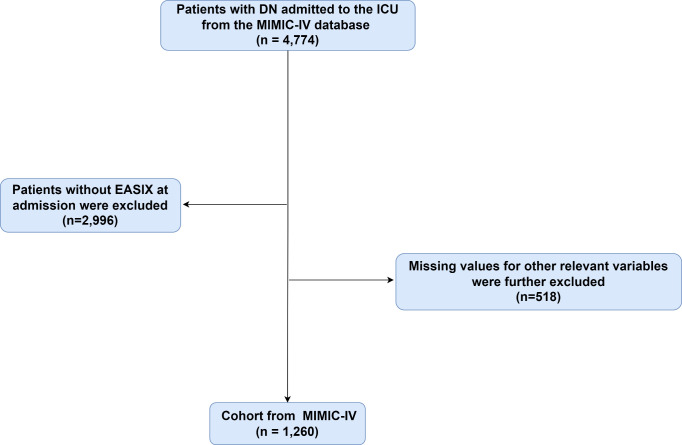
Flow chart of included patients through the trial.

Patients diagnosed with DN based on the International Classification of Diseases, 9th and 10th editions, were included. Exclusion criteria were as follows: (1) patients under 18 years old at the time of first admission; (2) patients with multiple ICU admissions, where only data from the first admission were used; and (3) patients with incomplete EASIX data on the first day of admission. In total, 1,260 patients were included, and they were grouped into three categories based on the tertiles of the EASIX index (Fig 1).

### 2.2. Data collection

We utilized PostgreSQL (version 13.7.2) and Navicate Premium (version 16) to extract data using Structured Query Language. The variables were categorized into four groups: (1) demographics, including age, gender, weight; (2) comorbidities, such as Atrial fibrillation, Sepsis, Heart failure, TIA, Stroke, Respiratory failure and Paraplegia; (3) laboratory indicators, comprising red blood cells (RBC), white blood cells (WBC), hemoglobin, platelets, serum sodium, serum creatinine, and fasting blood glucose (FBG); and (4) illness severity scores at admission, including the Acute Physiology Score III (APSIII), Simplified Acute Physiology Score II (SAPS-II), Oxford Acute Severity of Illness Score (OASIS), and Sepsis-related Organ Failure Assessment score (SOFA) [13,14]. Follow-up commenced on the admission date and concluded on the date of death. EASIX is calculated using the formula: serum lactate dehydrogenase (LDH) level (U/L) × creatinine level (mg/dL)/ platelet count (10^9/L), with all parameters being routine laboratory measurements [15].

### 2.3. Statistical analysis

Continuous variables were summarized as mean ± standard deviation (SD) or median with interquartile range, depending on data distribution, while categorical variables were presented as proportions. The Kolmogorov-Smirnov test was employed to evaluate the normality of continuous variables. AS EASIX had a skewed distribution, it was log2 transformed before analysis [15,16].

For normally distributed data, t-tests or ANOVA were utilized; for non-normally distributed data, the Mann-Whitney U test or Kruskal-Wallis test were applied. Kaplan-Meier analysis assessed the incidence of endpoints across EASIX levels, with differences evaluated using the log-rank test. Binary logistic regression identified factors associated with all-cause mortality risk. Cox proportional hazards models calculated hazard ratios (HR) and 95% confidence intervals (CI) for EASIX and endpoints, with adjustments in some models.

Confounders were selected based on p-values < 0.05 from univariate analyses, and clinically relevant variables were included in multivariable models: Crude model: unadjusted. Model 1: adjusted for sex, age, race, weight.Model 2: adjusted for sex, age, race, weight, CCI, GCS, OASIS, SAPS II, APS III, SOFA. Model 3: adjusted for sex, age, race, weight, CCI, GCS, OASIS, SAPS II, APS III, SOFA, Sodium, FBG, Serum creatinine, WBC, RBC, Platelet, Hemoglobin. Model 4: adjusted for sex, age, race, weight, CCI, GCS, OASIS, SAPS II, APS III, SOFA, Sodium, FBG, Serum creatinine, WBC, RBC, Platelet, Hemoglobin, TIA, Stroke, Sepsis, Paraplegia, Arterial fibrillation, Respiratory failure, Heart failure.

Additionally, a restricted cubic spline regression model with four knots was used to analyze the nonlinear relationship between baseline ICU EASIX and in-hospital all-cause mortality, treating EASIX as a continuous or ordinal variable (with the first tertile as the reference). And Boruta’s algorithm was used to rank the importance of potential risk factors for in-hospital mortality.

P-values for trends were derived from interquartile ranges. Stratification by sex, age (≤65 and >65 years), heart failure, atrial fibrillation, and sepsis assessed the consistency of EASIX’s prognostic value for the primary outcome. Interactions between EASIX and stratification variables were tested using likelihood ratio tests. All analyses were conducted using R software (version 4.0.2), and statistical significance was set at p < 0.05.

## 3. Results

This study included 1260 critically ill patients with DN. The median age was 72.38 years, and 35.71% (450) of the participants were male. Hospital and intensive care unit (ICU) mortality rates were 23.57% and 16.11%, respectively (Table 1).

**Table 1:** Characteristics and outcomes of participants categorized by the EASIX.

Variable	Total (n = 1260)	Q1 (n = 420)	Q2 (n = 419)	Q3 (n = 421)	p.value
Sex					<0.0001
Female	450(35.71)	185(44.05)	155(36.99)	110(26.13)	
Male	810(64.29)	235(55.95)	264(63.01)	311(73.87)	
Age	72.38 ± 11.06	73.37 ± 10.53	73.67 ± 10.74	70.12 ± 11.57	<0.0001
Race					0.01
Black	250(19.84)	71(16.90)	95(22.67)	84(19.95)	
Mexican American	1(0.08)	0(0.00)	0(0.00)	1(0.24)	
Other	312(24.76)	96(22.86)	90(21.48)	126(29.93)	
White	697(55.32)	253(60.24)	234(55.85)	210(49.88)	
Weight	84.00(71.48,101.73)	85.20(71.40,104.88)	84.00(71.25,103.15)	84.00(72.60,98.80)	0.58
Comorbidities					
Heart failure					0.13
No	1242(98.57)	418(99.52)	411(98.09)	413(98.10)	
Yes	18(1.43)	2(0.48)	8(1.91)	8(1.90)	
Respiratory failure					0.58
No	610(48.41)	212(50.48)	199(47.49)	199(47.27)	
Yes	650(51.59)	208(49.52)	220(52.51)	222(52.73)	
Arterial fibrillation					0.95
No	661(52.46)	218(51.90)	220(52.51)	223(52.97)	
Yes	599(47.54)	202(48.10)	199(47.49)	198(47.03)	
Paraplegia					0.60
No	1258(99.84)	419(99.76)	418(99.76)	421(100.00)	
Yes	2(0.16)	1(0.24)	1(0.24)	0(0.00)	
Sepsis					<0.0001
No	844(66.98)	295(70.24)	305(72.79)	244(57.96)	
Yes	416(33.02)	125(29.76)	114(27.21)	177(42.04)	
Stroke					0.02
No	1178(93.49)	381(90.71)	399(95.23)	398(94.54)	
Yes	82(6.51)	39(9.29)	20(4.77)	23(5.46)	
TIA					0.05
No	1257(99.76)	420(100.00)	416(99.28)	421(100.00)	
Yes	3(0.24)	0(0.00)	3(0.72)	0(0.00)	
Laboratory tests					
Hemoglobin, m/uL	9.51 ± 1.89	9.75 ± 1.97	9.44 ± 1.84	9.34 ± 1.83	<0.01
Platelet, K/uL	180.00(128.00,242.00)	226.00(179.75,299.00)	177.00(137.50,229.50)	131.00(75.00,183.00)	<0.0001
RBC, m/uL	3.28 ± 0.71	3.43 ± 0.73	3.25 ± 0.69	3.17 ± 0.69	<0.0001
WBC, K/uL	11.00(8.10,16.20)	10.80(8.30,15.50)	10.90(7.90,15.50)	11.60(7.80,17.80)	0.20
Serum creatinine	2.50(1.70,4.20)	1.60(1.30,2.30)	2.60(1.90,3.80)	4.40(3.00,6.30)	<0.0001
FBG, mg/dL	170.50(126.00,229.00)	170.50(130.00,227.25)	175.00(128.00,232.00)	168.00(120.00,223.00)	0.31
Sodium, mEq/L	138.30 ± 5.66	138.90 ± 5.49	138.74 ± 5.51	137.28 ± 5.84	<0.0001
SOFA	6.00(4.00,9.00)	4.00(3.00,6.00)	6.00(4.00,8.00)	9.00(7.00,12.00)	<0.0001
APSIII	54.00(44.00,67.00)	48.00(39.00,60.00)	52.00(43.00,62.00)	61.00(51.00,77.00)	<0.0001
SAPSII	42.00(35.00,52.00)	38.00(32.00,46.00)	42.00(35.00,50.00)	49.00(39.00,60.00)	<0.0001
OASIS	33.00(26.00,39.00)	31.00(26.00,38.00)	31.00(26.00,38.00)	35.00(29.00,41.00)	<0.0001
GCS	13.68 ± 2.60	13.74 ± 2.46	13.81 ± 2.37	13.49 ± 2.92	0.16
CCI	9.13 ± 1.84	9.07 ± 1.84	9.22 ± 1.80	9.10 ± 1.87	0.43
Events					
LOS ICU, days	2.73(1.50,5.68)	2.36(1.36,5.37)	2.65(1.48,5.17)	3.31(1.69,6.73)	<0.001
LOS hospital, days	10.79(6.13,19.79)	9.86(5.84,17.88)	10.56(6.28,18.00)	12.58(6.42,21.94)	0.04
Hospital mortality					<0.0001
Alive	963(76.43)	357(85.00)	335(79.95)	271(64.37)	
Death	297(23.57)	63(15.00)	84(20.05)	150(35.63)	
ICU mortality					<0.0001
Alive	1057(83.89)	388(92.38)	359(85.68)	310(73.63)	
Death	203(16.11)	32(7.62)	60(14.32)	111(26.37)	

Data are presented as mean (SE) or frequencies (percentages).

Abbreviation: EASIX, Endothelial Activation and Stress Index; BMI, body mass index; SOFA, sequential organ failure assessment; CCI, Charlson comorbidity index; APSIII, acute physiology score III; SAPSII, simplified acute physiological score II; OASIS, oxford acute severity of illness score; GCS, Glasgow coma scale; WBC, white blood cell; RBC, red blood cell; FBG, fasting blood glucose.

### 3.1. Baseline characteristics

Table 1 presents the baseline characteristics of critically ill DN patients stratified by EASIX tertiles. Patients in the highest EASIX tertile typically were younger, and exhibited a higher prevalence of respiratory failure and sepsis compared to those in the lower tertile. They also had higher levels of platelets, WBC, serum creatinine, SOFA, APS3, SAPSII, and OASIS. Notably, in-hospital mortality (15.00% vs. 35.63%, p < 0.0001) and ICU mortality (7.62% vs. 26.37%, p < 0.0001) were significantly higher in the highest EASIX tertile compared to the lower tertile.

Table 2 highlights differences in baseline characteristics between survivors and non-survivors during hospitalization. Non-survivors had lower weight, platelet count, glucose, sodium, SOFA, APS3, SAPSII, and OASIS. EASIX levels were significantly higher in the non-survivor group (3.03 vs. 2.20, P < 0.0001) compared to the survivor group.

**Table 2:** Baseline characteristics of Survivors and Non-survivors.

Variable	Total (n = 1260)	Alive (n = 963)	Death (n = 297)	p.value
EASIX	2.19(1.28,3.23)	2.02(1.19,3.03)	2.88(1.74,4.06)	<0.0001
Sex				0.29
Female	450(35.71)	352(36.55)	98(33.00)	
Male	810(64.29)	611(63.45)	199(67.00)	
Age	72.38 ± 11.06	71.84 ± 11.01	74.13 ± 11.08	<0.01
Race				0.04
Black	250(19.84)	197(20.46)	53(17.85)	
Mexican American	1(0.08)	1(0.10)	0(0.00)	
Other	312(24.76)	220(22.85)	92(30.98)	
White	697(55.32)	545(56.59)	152(51.18)	
Weight	84.00(71.48,101.73)	84.80(71.70,103.10)	81.90(71.30,99.90)	0.07
Comorbidities				
Heart failure				0.07
No	1242(98.57)	953(98.96)	289(97.31)	
Yes	18(1.43)	10(1.04)	8(2.69)	
Respiratory failure				<0.0001
No	610(48.41)	504(52.34)	106(35.69)	
Yes	650(51.59)	459(47.66)	191(64.31)	
Arterial fibrillation				0.10
No	661(52.46)	518(53.79)	143(48.15)	
Yes	599(47.54)	445(46.21)	154(51.85)	
Paraplegia				1.00
No	1258(99.84)	961(99.79)	297(100.00)	
Yes	2(0.16)	2(0.21)	0(0.00)	
Sepsis				<0.0001
No	844(66.98)	718(74.56)	126(42.42)	
Yes	416(33.02)	245(25.44)	171(57.58)	
Stroke				0.03
No	1178(93.49)	909(94.39)	269(90.57)	
Yes	82(6.51)	54(5.61)	28(9.43)	
TIA				1.00
No	1257(99.76)	961(99.79)	296(99.66)	
Yes	3(0.24)	2(0.21)	1(0.34)	
Laboratory tests				
Hemoglobin	9.51 ± 1.89	9.51 ± 1.86	9.50 ± 1.97	0.92
Platelet	180.00(128.00,242.00)	181.00(132.00,244.00)	176.00(117.00,238.00)	0.10
RBC	3.28 ± 0.71	3.29 ± 0.71	3.27 ± 0.73	0.68
WBC	11.00(8.10,16.20)	10.40(7.80,14.75)	14.40(9.30,20.60)	<0.001
Serum creatinine	2.50(1.70,4.20)	2.40(1.60,4.20)	2.80(2.00,4.20)	0.61
FBG	170.50(126.00,229.00)	168.00(126.00,224.50)	181.00(127.00,244.00)	0.01
Sodium	138.30 ± 5.66	138.26 ± 5.40	138.43 ± 6.43	0.69
SOFA	6.00(4.00,9.00)	6.00(4.00,8.00)	9.00(6.00,12.00)	<0.0001
APSIII	54.00(44.00,67.00)	51.00(42.00,61.00)	67.00(54.00,84.00)	<0.0001
SAPSII	42.00(35.00,52.00)	40.00(34.00,49.00)	52.00(42.00,63.00)	<0.0001
OASIS	33.00(26.00,39.00)	31.00(26.00,37.00)	38.00(31.00,44.00)	<0.0001
GCS	13.68 ± 2.60	13.87 ± 2.29	13.04 ± 3.34	<0.0001
CCI	9.13 ± 1.84	9.03 ± 1.83	9.45 ± 1.84	<0.001

Data are presented as mean (SE) or frequencies (percentages).

Abbreviation: BMI, body mass index; SOFA, sequential organ failure assessment; CCI, Charlson comorbidity index; APSIII, acute physiology score III; SAPSII, simplified acute physiological score II; OASIS, oxford acute severity of illness score; GCS, Glasgow coma scale; WBC, white blood cell; RBC, red blood cell; FBG, fasting blood glucose.

### 3.2. EASIX and mortality

#### 3.2.1. Machine learning highlights the role of EASIX in mortality prediction.

**Fig 2:**
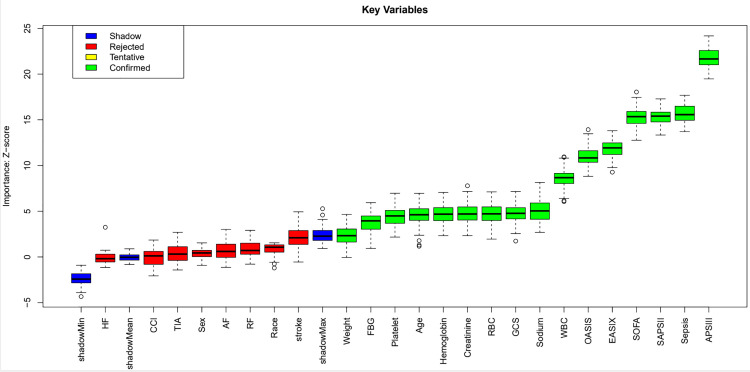
Boruta’s algorithm ranks the importance of potential risk factors for in-hospital mortality. Abbreviation: BMI, body mass index; SOFA, sequential organ failure assessment; CCI, Charlson comorbidity index; APSIII, acute physiology score III; SAPSII, simplified acute physiological score II; OASIS, oxford acute severity of illness score; GCS, Glasgow coma scale; WBC, white blood cell; RBC, red blood cell; FBG, fasting blood glucose; HF, Heart failure; AF, Arterial fibrillation; RF, Respiratory failure.

The Fig 2 illustrates the variable importance ranking determined by the Boruta algorithm. The x-axis represents the candidate variables, while the y-axis corresponds to the Importance Z-scores of these variables. Different colors in the boxplots indicate the significance categories of the variables: blue represents “Shadow” variables, red indicates “Rejected” variables, yellow corresponds to “Tentative” variables, and green denotes “Confirmed” variables. Notably, the EASIX variable has a high Z-score and is categorized as a “Confirmed” variable, signifying its significant importance in predicting in-hospital mortality. Other “Confirmed” variables, such as the SOFA score, SAPSII score, and APSIII score, further highlight their critical roles in the prediction model.

#### 3.2.2. Kaplan–Meier survival analysis curves for all-cause mortality.

**Fig 3:**
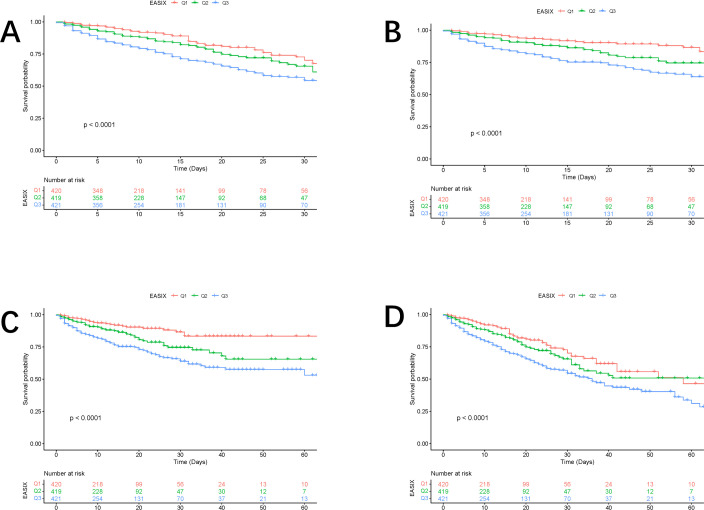
Kaplan–Meier curves showing the cumulative probability of mortality. **(A)** Death within 30 days in the ICU. **(B)** Death within 30 days in the hospital. **(C)** Death within 60 days in the ICU. **(D)** Death within 60 days in the hospital.

Kaplan-Meier survival curves, as depicted in Fig 3, were utilized to compare the incidence of the primary outcome across groups stratified by EASIX tertiles. Patients with higher EASIX exhibited a greater risk of hospitalization and ICU mortality. Additionally, the differences observed between 30 and 60 days were statistically significant (P < 0.0001).

#### 3.2.3. Cox proportional hazard ratios for all-cause mortality.

Cox proportional hazards analysis was employed to examine the relationship between EASIX and in-hospital mortality. EASIX was identified as a significant risk factor in various models. In the unadjusted model, the HR was 1.16 (95% CI 1.09–1.23, P < 0.0001). In the partially adjusted model, the HR was 1.20 (1.13–1.28, P = 0.01), and in the fully adjusted model, it was 1.14 (1.03–1.26, P = 0.01), indicating its significance as a continuous variable.

When EASIX was treated as a categorical variable, patients in the highest tertile exhibited a significantly higher risk of in-hospital death across four Cox proportional hazards models: unadjusted model [HR, 2.08 (95% CI 1.55–2.79), P < 0.0001], partially adjusted model [HR, 1.95 (95% CI 1.25–3.03), P = 0.003], and fully adjusted model [HR, 1.96 (95% CI 1.26–3.06), P = 0.003]. Similar findings were observed in multivariate analyses of EASIX and ICU mortality (Table 3).

**Table 3:** Cox regression analysis of EASIX levels and mortality in DN patients.

Categories	Crude model		Model 1		Model 2		Model 3		Model 4	
	95%CI	P	95%CI	P	95%CI	P	95%CI	P	95%CI	P
Hospital mortality										
Continuous variable per unit	1.16(1.09,1.23)	<0.0001	1.2(1.13, 1.28)	<0.0001	1.05(0.97, 1.13)	0.24	1.14(1.04, 1.26)	0.01	1.14(1.03, 1.26)	0.01
Tertile										
Q1	ref		ref		ref		ref		ref	
Q2	1.36(0.98,1.89)	0.06	1.33(0.95, 1.87)	0.09	1.07(0.76, 1.51)	0.70	1.23(0.85, 1.79)	0.27	1.27(0.87, 1.84)	0.22
Q3	2.08(1.55,2.79)	<0.0001	2.35(1.73, 3.19)	<0.0001	1.31(0.91, 1.88)	0.14	1.95(1.25, 3.03)	0.003	1.96(1.26, 3.06)	0.003
p for trend		<0.0001		<0.0001		0.12		0.003		0.002
ICU mortality										
Continuous variable per unit	1.2(1.12,1.29)	<0.0001	1.27(1.17, 1.37)	<0.0001	1.06(0.96, 1.17)	0.27	1.2(1.06, 1.35)	0.005	1.17(1.04, 1.33)	0.01
Tertile										
Q1	ref		ref		ref		ref		ref	
Q2	1.81(1.18,2.79)	0.01	1.83(1.17, 2.85)	0.01	1.29(0.81, 2.04)	0.28	1.64(0.99, 2.69)	0.05	1.62(0.98, 2.67)	0.06
Q3	2.78(1.87,4.12)	<0.0001	3.23(2.15, 4.87)	<0.0001	1.44(0.89, 2.32)	0.14	2.48(1.36, 4.50)	0.003	2.44(1.35, 4.42)	0.003
p for trend		<0.0001		<0.0001		0.15		0.003		0.003

Crude model: unadjusted.

Model 1: adjusted for sex, age, race, weight.

Model 2: adjusted for sex, age, race, weight, CCI, GCS, OASIS, SAPS II, APS III, SOFA.

Model 3: adjusted for sex, age, race, weight, CCI, GCS, OASIS, SAPS II, APS III, SOFA, Sodium, FBG, Serum creatinine, WBC, RBC, Platelet, Hemoglobin.

Model 4: adjusted for sex, age, race, weight, CCI, GCS, OASIS, SAPS II, APS III, SOFA, Sodium, FBG, Serum creatinine, WBC, RBC, Platelet, Hemoglobin, TIA, Stroke, Sepsis, Paraplegia, Arterial fibrillation, Respiratory failure, Heart failure.

Abbreviation: BMI, body mass index; SOFA, sequential organ failure assessment; CCI, Charlson comorbidity index; APSIII, acute physiology score III; SAPSII, simplified acute physiological score II; OASIS, oxford acute severity of illness score; GCS, Glasgow coma scale; WBC, white blood cell; RBC, red blood cell; FBG, fasting blood glucose.

#### 3.2.4. RCS for all-cause mortality.

**Fig 4:**
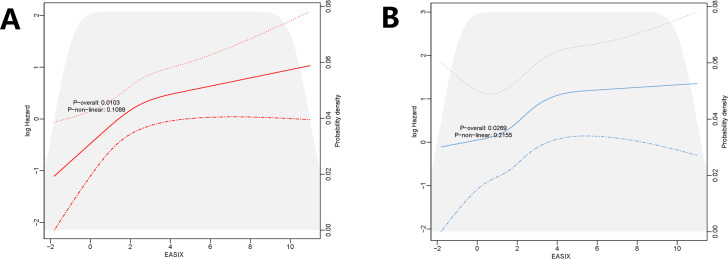
RCS for all-cause mortality. **(A)** In-hospital mortality. **(B)** In-ICU mortality.

The RCS regression model showed that with increasing EASIX, the risk of in-hospital mortality increased nonlinearly (P nonlinear = 0.1089), and the risk of ICU mortality increased linearly (P nonlinear = 0.2155) (Fig 4).

### 3.3. Subgroup analysis

**Fig 5:**
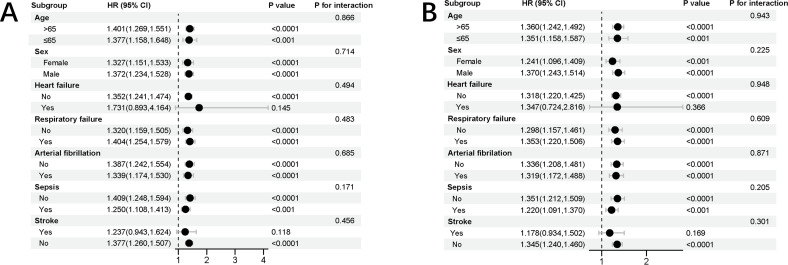
Forest plots of hazard ratios for mortality across subgroups. **(A)** Hospital mortality. **(B)** ICU mortality.

Fig 5 presents a stratified analysis of the relationship between EASIX and mortality, emphasizing its predictive value for ICU and hospitalization outcomes across various patient groups. Higher EASIX scores were associated with increased ICU and hospitalization mortality. This correlation was especially notable in older patients (≥65 years), male patients, and those with severe comorbidities such as cardiovascular disease or respiratory failure, indicating that these groups are particularly susceptible.

We observed that individuals without a history of stroke were at a higher risk of in-hospital and ICU mortality. Additionally, a positive history of heart failure was significantly associated with increased ICU mortality.

## 4. Discussion

This study explored the relationship between EASIX scores and short-term all-cause mortality (30-day and 60-day) in critically ill patients with DN. Using the MIMIC-IV database, we found that higher EASIX scores were independently linked to increased mortality in this group, indicating that EASIX could serve as a novel prognostic biomarker for critically ill DN patients.

The findings of this study emphasize the importance of endothelial activation and stress in the progression and prognosis of DN. Endothelial dysfunction, a key feature of DN, is driven by chronic inflammation and oxidative stress, which are intensified in critically ill patients [17]. The EASIX score, which includes LDH, creatinine, and platelet count, offers a clinically practical measure of endothelial damage and systemic stress. Patients in the highest EASIX tertile had significantly higher ICU and in-hospital mortality rates compared to those in the lower tertiles. Kaplan-Meier survival curves and Cox proportional hazard models consistently showed that Higher EASIX scores are associated with increased short-term mortality in critically ill DN patients, even after adjusting for relevant confounders. 

In the context of COVID-19, EASIX has been identified as a strong predictor of mortality. A study involving hospitalized COVID-19 patients found that those with higher EASIX scores had significantly poorer overall survival compared to those with lower scores. This finding highlights the potential of EASIX as a universal prognostic tool for both hematological and non-hematological COVID-19 patients [18].

Similarly, in patients undergoing allogeneic hematopoietic stem cell transplantation, EASIX has been validated as a predictor of non-relapse mortality. A prospective study confirmed that an EASIX score of 3 or higher was associated with more than a twofold increased risk of treatment-related mortality, emphasizing its value in identifying high-risk patients prior to transplantation [19].

In hypertensive individuals, EASIX has been associated with both all-cause and cardiovascular mortality. A nationwide study found that each 1-unit increase in EASIX corresponded to a significant rise in mortality risk, suggesting its potential as a predictive biomarker for hypertensive patients in clinical practice [20]. In sepsis, EASIX has been linked to an increased mortality risk. A retrospective cohort study demonstrated that higher EASIX scores were associated with a greater risk of 28-day and 90-day all-cause mortality in septic patients, highlighting its relevance in critical care settings [16].

DN is a common complication in diabetic patients, with a complex pathogenesis driven by inflammation and oxidative stress as key factors [21,22]. Oxidative stress arises from an imbalance between the production of reactive oxygen species (ROS) and the body’s antioxidant defenses, playing a crucial role in the onset and progression of DN [23,24]. In DN, oxidative stress directly damages glomerular and tubular cells, exacerbating renal injury by activating inflammatory responses. It further amplifies inflammation through processes such as lipid peroxidation, protein oxidation, and DNA damage [25]. Additionally, oxidative stress can upregulate the expression of pro-inflammatory cytokines by activating signaling pathways like nuclear factor κB, thus worsening inflammatory responses [26].

Inflammation plays a critical role in the progression of DN. Research has shown that pro-inflammatory cytokines, such as tumor necrosis factor-α and interleukin-6, are significantly elevated in diabetic patients. These cytokines contribute to renal function deterioration by promoting glomerulosclerosis and tubulointerstitial fibrosis [27,28]. Furthermore, inflammation can induce oxidative stress, creating a vicious cycle that accelerates the progression of DN [29].

Oxidative stress and inflammation are key factors contributing to endothelial dysfunction, and their relationship has been extensively studied across various diseases. Oxidative stress arises from an imbalance between oxidants and antioxidants, leading to the overproduction of ROS and reactive nitrogen species, which can cause cellular damage and dysfunction. Inflammation, on the other hand, is the body’s response to injury or infection, marked by the activation of immune cells and the release of inflammatory cytokines. These two factors interact in a complex manner: oxidative stress can trigger inflammatory responses, while inflammation can amplify oxidative stress, creating a vicious cycle [30–35].

During the process of endothelial dysfunction, oxidative stress and inflammation work together to cause damage and dysfunction of endothelial cells. Oxidative stress directly damages the lipids, proteins, and DNA of endothelial cell membranes by increasing ROS production, leading to apoptosis and necrosis. Additionally, oxidative stress can also impair the vasodilatory function of endothelial cells by inhibiting the biosynthesis of nitric oxide and reducing its bioavailability [31–33].

The EASIX score, by reflecting endothelial stress, captures these underlying pathological processes and provides a reliable measure of disease severity.

The nonlinear relationship between EASIX and in-hospital mortality, as observed in the restricted cubic spline regression, suggests that even moderate increases in EASIX may significantly impact mortality risk. This is consistent with prior research in other settings, such as CAR-T cell therapy and traumatic brain injury, where EASIX has been shown to predict adverse outcomes [10,36]. Furthermore, subgroup analyses revealed that the prognostic value of EASIX was particularly evident in older patients, males, and those with severe comorbidities, such as cardiovascular disease or respiratory failure. These findings suggest that certain patient subgroups are more vulnerable to the adverse effects of endothelial dysfunction and may benefit from targeted monitoring and interventions.

## 5. Limitations

While this study provides important insights, it has several limitations. First, the retrospective design precludes causal inferences, and residual confounding cannot be ruled out despite rigorous statistical adjustments. Second, the study was conducted using data from a single-center database, which may limit the generalizability of the findings to other populations or healthcare settings. Third, EASIX values were only assessed on the first day of ICU admission, and dynamic changes in endothelial function over time were not captured. Future studies should evaluate the temporal trends of EASIX and their impact on outcomes. Lastly, additional biomarkers of endothelial dysfunction and inflammation could be incorporated to improve the predictive accuracy of the EASIX score.

## 6. Conclusions

Higher EASIX scores are associated with increased short-term mortality in critically ill DN patients. These findings suggest that EASIX could serve as a valuable biomarker for risk stratification and personalized management of DN patients in the ICU.

## Supporting information

S1 File Supplementary data set. (XLSX)

## Abbreviations

DN: Diabetic nephropathy
EASIX: Endothelial Activation and Stress Index
